# Development and Validation of an Algorithm to Identify Patients with Multiple Myeloma Using Administrative Claims Data

**DOI:** 10.3389/fonc.2016.00224

**Published:** 2016-10-27

**Authors:** Nicole Princic, Chris Gregory, Tina Willson, Maya Mahue, Diana Felici, Winifred Werther, Gregory Lenhart, Kathleen A. Foley

**Affiliations:** ^1^Truven Health, Cambridge, MA, USA; ^2^Onyx Pharmaceuticals Inc., An Amgen Subsidiary, San Francisco, CA, USA

**Keywords:** multiple myeloma, administrative claims, electronic medical records, algorithm

## Abstract

**Purpose:**

The objective was to expand on prior work by developing and validating a new algorithm to identify multiple myeloma (MM) patients in administrative claims.

**Methods:**

Two files were constructed to select MM *cases* from MarketScan Oncology Electronic Medical Records (EMR) and *controls* from the MarketScan Primary Care EMR during January 1, 2000–March 31, 2014. Patients were linked to MarketScan claims databases, and files were merged. Eligible cases were age ≥18, had a diagnosis and visit for MM in the Oncology EMR, and were continuously enrolled in claims for ≥90 days preceding and ≥30 days after diagnosis. Controls were age ≥18, had ≥12 months of overlap in claims enrollment (observation period) in the Primary Care EMR and ≥1 claim with an ICD-9-CM diagnosis code of MM (203.0×) during that time. Controls were excluded if they had chemotherapy; stem cell transplant; or text documentation of MM in the EMR during the observation period. A split sample was used to develop and validate algorithms. A maximum of 180 days prior to and following each MM diagnosis was used to identify events in the diagnostic process. Of 20 algorithms explored, the baseline algorithm of 2 MM diagnoses and the 3 best performing were validated. Values for sensitivity, specificity, and positive predictive value (PPV) were calculated.

**Conclusion:**

Three claims-based algorithms were validated with ~10% improvement in PPV (87–94%) over prior work (81%) and the baseline algorithm (76%) and can be considered for future research. Consistent with prior work, it was found that MM diagnoses before and after tests were needed.

## Introduction

Cancer research using secondary data sources requires accurate identification of patients with specific cancer diagnoses. A recent review of cancer studies using secondary data sources, found 36% used just a single claim with a cancer diagnosis to identify the study sample while only 6.5% used a validated algorithm (1). The diagnostic process for cancer involves many laboratory blood tests, imaging, and surgical biopsies, therefore just one or two health service claims with the cancer diagnosis code may not be sufficient for correct identification of a cancer population.

Multiple myeloma (MM) is a hematologic cancer that leads to the accumulation of malignant plasma cells in the bone marrow and over production of monoclonal proteins in the serum or urine (2). It is the second most common hematologic malignancy, accounting for 12% of hematologic cancers worldwide (3). Recent advances in treatment have extended survival but prognosis is still poor with an overall median survival of 4–7 years from initial diagnosis (4).

Patients with MM are diagnosed by pathological changes in the blood and symptoms of hypercalcemia, renal dysfunction, anemia, and bone pain from lesions (referred to as the CRAB criteria) (5–7). MM is part of a spectrum of monoclonal plasma cell disorders, leading to increased complexity in diagnosing the disease. Patients with suspected MM are typically identified as having monoclonal gammopathy of undetermined significance (MGUS), smoldering MM (SMM), or active MM, with additional testing needed to determine a definitive diagnosis (8–12). MGUS, defined as having less than 10% plasma cells in the bone marrow and a monoclonal protein level less than 3 g/dL (5, 8), represents the least pathologically advanced disorder along this spectrum, is asymptomatic, and requires no therapeutic intervention. Patients with MGUS progress to active MM at a rate of ~0.26–12% per year (depending on tumor type) and many patients never progress (9, 10, 13).

Smoldering MM, often referred to as “early MM,” has a risk of progressing of 10% per year for the first 5 years, 3% per year for the next 5 years, and 1–2% per year for the next 10 years (11, 12). Patients with SMM have an excess of monoclonal protein in the blood and urine but are asymptomatic. Definitive diagnosis of SMM includes a monoclonal protein level of at least 3 g/dL or the proportion of plasma cells in the bone marrow is at least 10% (5, 8). SMM patients are monitored for disease progression with treatment initiated only after progression. However, recent literature suggests earlier treatment may be beneficial (8, 14).

Given the complexity of diagnosing MM, it is possible that patients may be misdiagnosed during the diagnostic process. MM diagnoses can appear on diagnostic claims to rule out disease, can result in misclassification of individuals as having MM, when they actually have MGUS or a different cancer. Prior research in MM using claims data has relied on identifying a treated population (15–17). While the strategy of requiring both appropriate diagnosis codes and disease-specific treatments can better identify true cases, this approach excludes the untreated population from research questions. Recent work to develop and validate an algorithm identifying MM patients in administrative claims used the SEER Tumor Registry (18). At least two diagnoses before and after a diagnostic procedure code within 90 days were needed to achieve a positive predictive value (PPV) of 81% and a sensitivity of 73% (18). The primary objective of this analysis was to expand and improve on prior work by developing and validating algorithms with better PPV and sensitivity. The secondary objective was to determine if an algorithm could be created to identify patients with SMM or untreated MM.

## Methods

### Data Sources

This study utilized MM cases and controls from four Marketscan^®^ databases: two MarketScan Electronic Medical Records (EMR) Databases (Oncology EMR and Primary Care EMR) and two MarketScan administrative claims databases (Commercial and Medicare Supplemental). The Commercial and Medicare Supplemental claims databases includes employer- and health plan-sourced medical and outpatient pharmacy data for ~41 million enrollees per year, linked by a unique blinded identifier across the continuum of care. All databases were de-identified in compliance with Health Insurance Portability and Accountability Act (HIPAA) regulations. Patients eligible for the analysis were present in the Claims-Oncology EMR Linked Dataset or the Claims-Primary Care EMR Linked Dataset, which combine the clinical detail from EMR and the claims-level details of all provider visits, diagnoses, procedures, and medications needed for algorithm development.

### Patient Selection

As an initial step, two separate files were constructed to select cases (true MM patients; “gold standard”) from the MarketScan Oncology EMR database linked to the MarketScan Commercial and Medicare claims databases and controls (patients known to be absent of MM) from the MarketScan Primary Care EMR database linked to the MarketScan claims databases during January 1, 2000–March 31, 2014 (study period).

Inclusion criteria for MM cases were, age 18 years or older, have a diagnosis and a clinic visit date for MM in the Oncology EMR and be continuously enrolled in the claims database for at least 90 days preceding and 30 days following the EMR diagnosis. MM cases were identified as incident if there were no claims for chemotherapy treatment or administration associated with an MM diagnosis prior to the EMR date of diagnosis.

**Figure 1:**
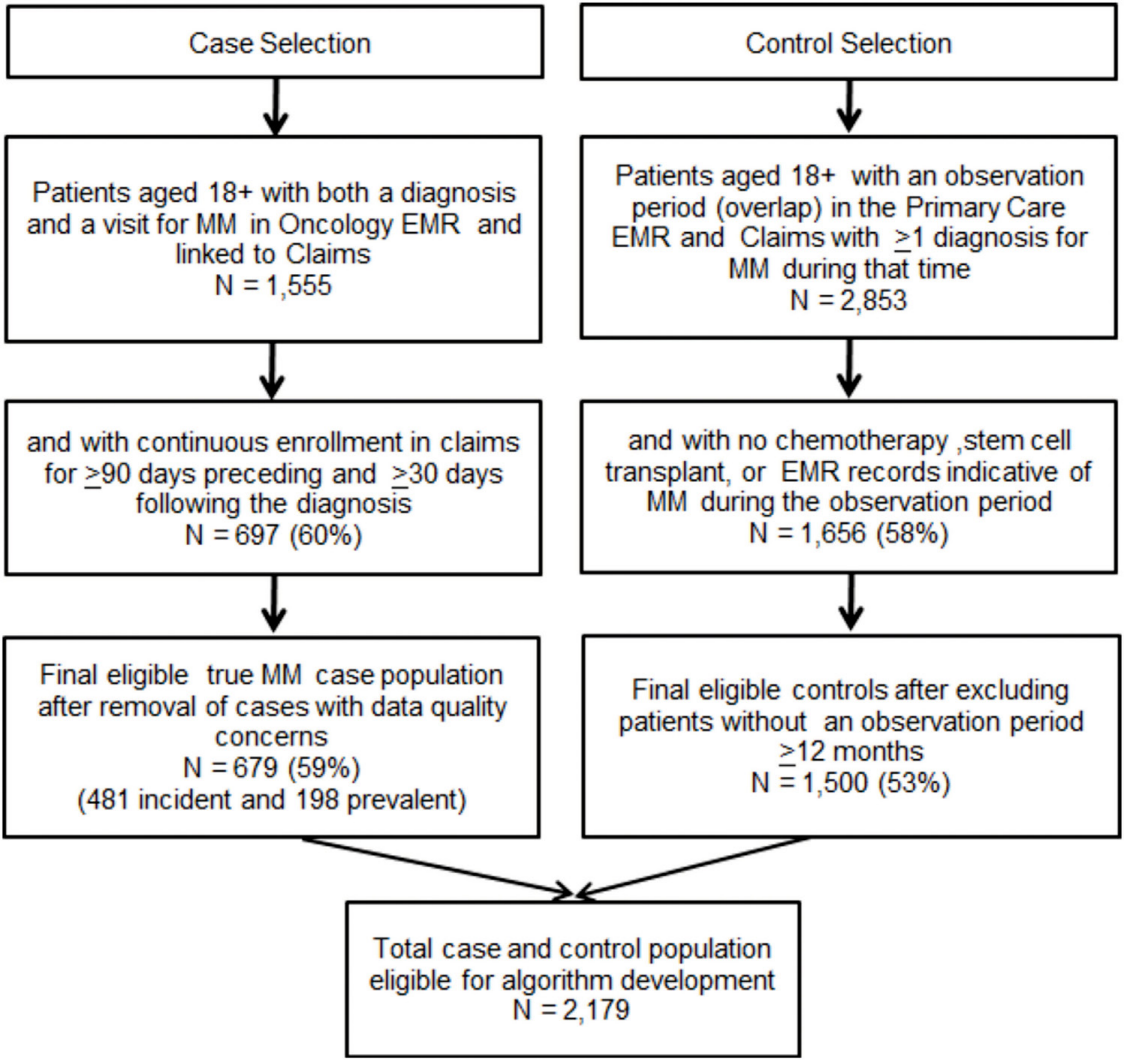
**Case and control selection**.

Inclusion criteria for MM controls were, age 18 years or older, at least 12 months of overlap in enrollment (observation period) in both the Primary Care EMR and claims and have at least 1 claim with an ICD-9-CM diagnosis code of MM (203.0×) during that time. To ensure, controls did not have MM during the observation period, patients meeting any of the following criteria were excluded: (1) a chemotherapy treatment or administration claim associated with an MM diagnosis, (2) a stem cell transplant procedure claim, and (3) evidence of MM in the primary care EMR identified through review of all primary care records with any text documentation of MM. Following selection of cases and controls, the two files were merged for algorithm development. Figure 1 depicts the case and control selection process.

### Algorithm Development

**Figure 2:**
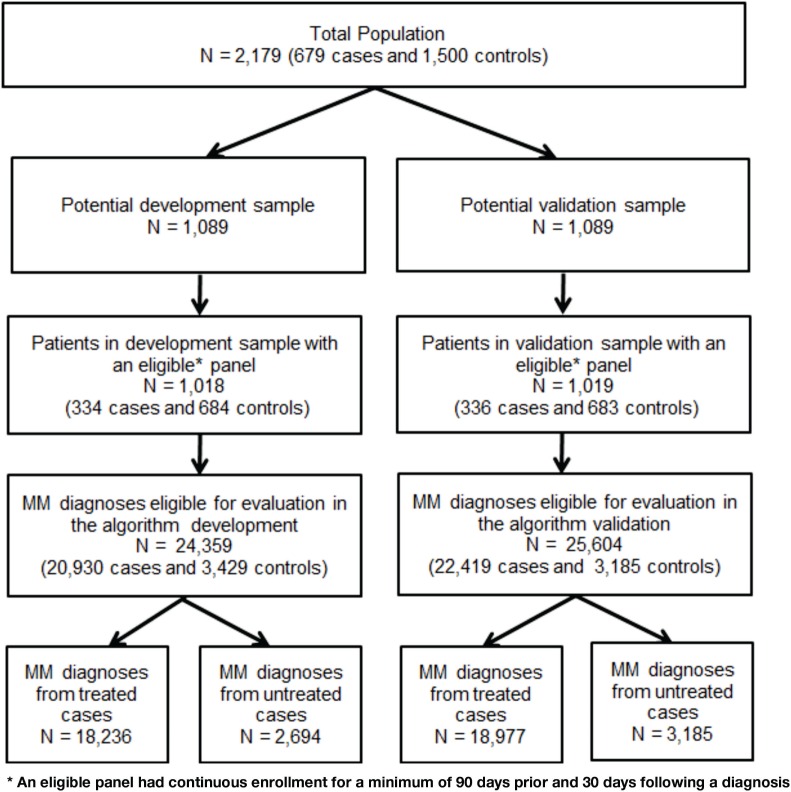
**Development and validation sample split**.

The file was randomly split in half with the first half used to develop the algorithm (development sample) and the second half to validate the algorithm (validation sample). A panel file was constructed to test algorithms on all MM diagnoses in the claims data for cases and controls captured during the study period. A diagnosis was eligible for inclusion in the panel as an index event if there was continuous enrollment for a minimum of 90 days prior and 30 days following the diagnosis. A maximum of 180 days before and after each eligible diagnosis was used to identify tests, treatments, and symptoms used in the MM diagnostic process. Figure 2 portrays the process of splitting the sample and identification of eligible claims diagnoses.

All variables used in the algorithm were claims based and identified using enrollment records, service dates, International Classification of Diseases, 9th Revision, Clinical Modification (ICD-9-CM) codes, Current Procedural Technology 4th edition (CPT-4^®^) codes, Healthcare Common Procedure Coding System (HCPCS) codes, and National Drug Codes (NDCs). Using the development sample, each MM diagnosis was labeled first as a case or a control. Diagnoses from cases were then divided further to analyze untreated and treated patients. Data were explored for differences in patterns (combinations, timing, and sequences of events) between cases and controls through descriptive analysis and review of patient profiles. Table 1 contains the average annual number of tests, MM diagnoses, and days of chemotherapy treatment per patient tabulated by MM status and the presence or absence of MM chemotherapy treatment in the development sample. There were no notable patterns in the development sample that differentiated untreated cases from controls.

**Table 1:** **Average annual number of diagnostic tests, MM diagnoses, and days of chemotherapy treatment per patient tabulated by true MM status and presence or absence of chemotherapy treatment using development sample**.

Patient status	*N*	Protein electrophoresis	Quantitative immunoglobulin	Serum free light chain	Serum albumin	Serum beta 2-microglobulin	Bone marrow	X-ray	Lactate dehydrogenase	MM diagnoses	Days of chemo
Controls	684	1.20	0.68	0.34	0.05	0.28	0.93	0.27	0.63	3	–
Incident cases: untreated	54	2.25	1.36	1.12	0.05	0.84	0.88	0.60	0.77	8	–
Prevalent cases: untreated	18	3.80	1.31	1.00	0.04	0.65	1.03	0.47	0.62	18	–
Incident cases: treated	182	4.80	2.69	2.23	0.14	1.27	1.67	1.00	2.50	39	240
Prevalent cases: treated	80	5.37	3.09	3.17	0.09	1.93	0.95	0.67	2.85	34	202

More than 20 different algorithms were created in the development sample and applied to both the total population and then separately to the untreated cases. Algorithms were derived using clinical expertise in the MM disease area and by review of patterns in the descriptive results. The best performing algorithms (i.e., highest specificity and sensitivity) in the development sample were applied to the validation sample where final values for sensitivity, specificity, and PPV were calculated. Sensitivity and specificity were calculated directly using the algorithm results. Since the cases and controls were identified from two different databases, the PPV could not be calculated directly, but was derived applying the algorithm to a large general population claims (Marketscan) database and identifying the percent with a MM diagnosis flagged. This percentage was used in the following formula with the sensitivity and specificity of the algorithm, to calculate the PPV in MarketScan [PPV = (sensitivity × (% flagged + specificity − 1))/(% flagged × (sensitivity + specificity − 1))]. The derivation of the formula can be found in Supplementary Material.

## Results

From the 336 cases and the 683 controls who comprised the validation sample, controls had 3,185 and cases had 22,419 (3,442 untreated; 18,977 treated) MM diagnoses available for evaluation. Demographic characteristics were similar for cases and controls with the mean age ~65 years and even gender and payer distribution (~50% male and ~50% commercially insured).

Multiple myeloma cases had a larger proportion of patients with diagnostic tests, more diagnostics tests, a larger proportion of the majority of symptoms, and more claims with an MM diagnosis code compared with controls up to 180 days prior to each index diagnosis. Compared with controls, cases had a larger proportion of patients with corticosteroid treatment (72 vs. 26%), anemia treatment with blood transfusion or erythropoiesis-stimulating agents (30 vs. 14%), osteoporosis treatment with bisphosphonates (60 vs. 14%), renal failure (22 vs. 15%), anemia diagnoses (36 vs. 29%), and skeletal related events (13 vs. 9%) during the 180 days prior to each index diagnosis. Additionally, although cases had more claims with a MM diagnosis code prior to each index diagnosis (36.7), controls still averaged 8.1.

Multiple myeloma untreated cases had similar characteristics (tests, symptoms, number of diagnoses) prior to each diagnosis as controls. Table 2 presents descriptive results of symptoms, tests, treatments, and diagnoses used in the algorithm development stratified by MM diagnoses from cases (treated and untreated) and controls.

**Table 2:** **MM symptoms and clinical characteristics around each multiple myeloma diagnosis in the algorithm validation sample**.

	Control^a^ MM diagnoses *N* = 3,185	Cases MM diagnoses *N* = 22,419	Case: untreated MM diagnoses *N* = 3,442	Case: treated MM diagnoses *N* = 18,977
**MM diagnoses**				
MM dx prior^b^ to indexed diagnosis (%)	100.0	100.0	100.0	100.0
Number of MM dx prior^b^; mean (SD)	8.1 (10.4)	36.7 (29.8)	14.9 (12.7)	40.7 (30.3)
MM dx following^c^ indexed diagnosis (%)	73.9	99.1	96.0	99.7
Number of MM dx following; mean (SD)	6.9 (10.3)	34.7 (29.1)	12.3 (10.8)	38.8 (29.5)
**Treatment**				
Chemo prior^b^ to or on indexed diagnosis (%)	0.0	76.9	0.0	90.8
Chemo following^c^ indexed diagnosis (%)	0.0	73.5	0.0	86.9
Corticosteroids prior to indexed diagnosis (%)	26.1	71.8	28.3	79.7
Corticosteroids following indexed diagnosis (%)	22.8	61.0	24.4	67.6
**Symptoms prior^b^ to indexed diagnosis**				
Monocloncal gammopathy (%)	19.3	11.3	4.1	12.6
Other malignancies (%)	18.6	18.2	12.4	19.2
Anemia diagnosis (%)	29.1	36.1	24.8	38.2
Anemia treatment (%)	13.8	29.8	18.0	31.9
Osteoporosis diagnosis (%)	3.4	1.6	0.9	1.7
Osteoporosis treatment (%)	13.6	60.3	54.2	61.4
Skeletal-related event (%)	9.3	12.6	7.6	13.6
Bone pain/lesions (%)	7.2	9.2	5.0	10.0
Fatigue (%)	9.7	11.5	5.4	12.6
Shortness of breath (%)	7.6	9.8	9.6	9.9
Chest pain (%)	13.8	16.0	14.3	16.3
Peripheral neuropathy (%)	4.8	2.9	3.0	2.9
Renal failure (%)	15.2	21.9	11.1	23.9
Hypercalcemia (%)	1.0	4.9	2.7	5.3
Pneumonia (%)	7.7	7.3	4.6	7.7
Herpes zoster (%)	1.3	2.5	2.1	2.6
Urinary tract/kidney infections (%)	9.1	5.1	6.6	4.8
**Tests prior^b^ to indexed diagnosis**				
Urine or serum protein electrophoresis (%)	55.0	70.3	58.8	72.3
Quantitative immunoglobulin levels (%)	39.3	53.7	48.4	54.7
Serum free light chain assay (%)	25.6	44.4	33.5	46.4
Serum albumin (%)	2.3	7.7	2.5	8.7
Serum beta 2-microglobulin (%)	24.3	37.1	31.7	38.1
Bone marrow aspirate or biopsy (%)	46.6	59.8	34.3	64.4
X-ray, skeletal survey/complete (%)	24.1	41.9	28.0	44.5
Lactate dehydrogenase test (%)	28.2	42.6	31.5	44.6
Number of each test; mean (SD)				
Urine or serum protein electrophoresis	1.3 (1.7)	3.7 (5.1)	2.2 (2.8)	4.0 (5.4)
Quantitative immunoglobulin levels	0.8 (0.3)	2.2 (3.5)	1.3 (1.8)	2.4 (3.7)
Serum free light chain assay	0.5 (1.0)	1.9 (4.1)	0.8 (1.4)	2.1 (4.3)
Serum albumin	0.0 (0.2)	0.4 (1.9)	0.0 (0.3)	0.5 (2.1)
Serum beta 2-microglobulin	0.4 (0.9)	1.1 (2.2)	0.8 (1.5)	1.1 (2.3)
Bone marrow aspirate or biopsy	0.8 (1.2)	1.1 (1.3)	0.5 (0.9)	1.2 (1.3)
X-ray, skeletal survey/complete	0.3 (0.5)	0.5 (0.7)	0.3 (0.5)	0.5 (0.7)
Lactate dehydrogenase test	0.7 (2.2)	2.6 (5.8)	1.0 (2.0)	2.9 (6.2)
Number of different symptom types (mean, SD)	1.9 (1.8)	2.6 (1.8)	1.9 (1.6)	2.7 (2.0)
Number of different tests types (mean, SD)	2.5 (2.0)	3.6 (2.3)	2.7 (2.2)	3.7 (2.3)

*^a^Control diagnoses are inclusive of (a) MM dx from control population (b) incident case MM dx before they became a case (i.e., prior to the confirmed dx date in the EMR and have no chemotherapy for MM)*.

*^b^180 days prior to indexed diagnosis*.

*^c^180 days following indexed diagnosis*.

Of 20 algorithms explored in the development sample, the baseline algorithm of 2 MM diagnoses and the 3 algorithms with highest specificity and sensitivity were run in the validation sample. All algorithms started at the earliest MM diagnosis date in each patient’s observation window and evaluated the time period during the 180 days prior and the 180 days following the diagnosis. If the patient did not meet the algorithm requirements at the first diagnosis, the next diagnosis was evaluated for inclusion. A patient was flagged by the algorithm at the earliest MM diagnosis who met all requirements. This approach was used to maximize the potential for capturing patients as early in the diagnostic process as possible.

**Figure 3:**
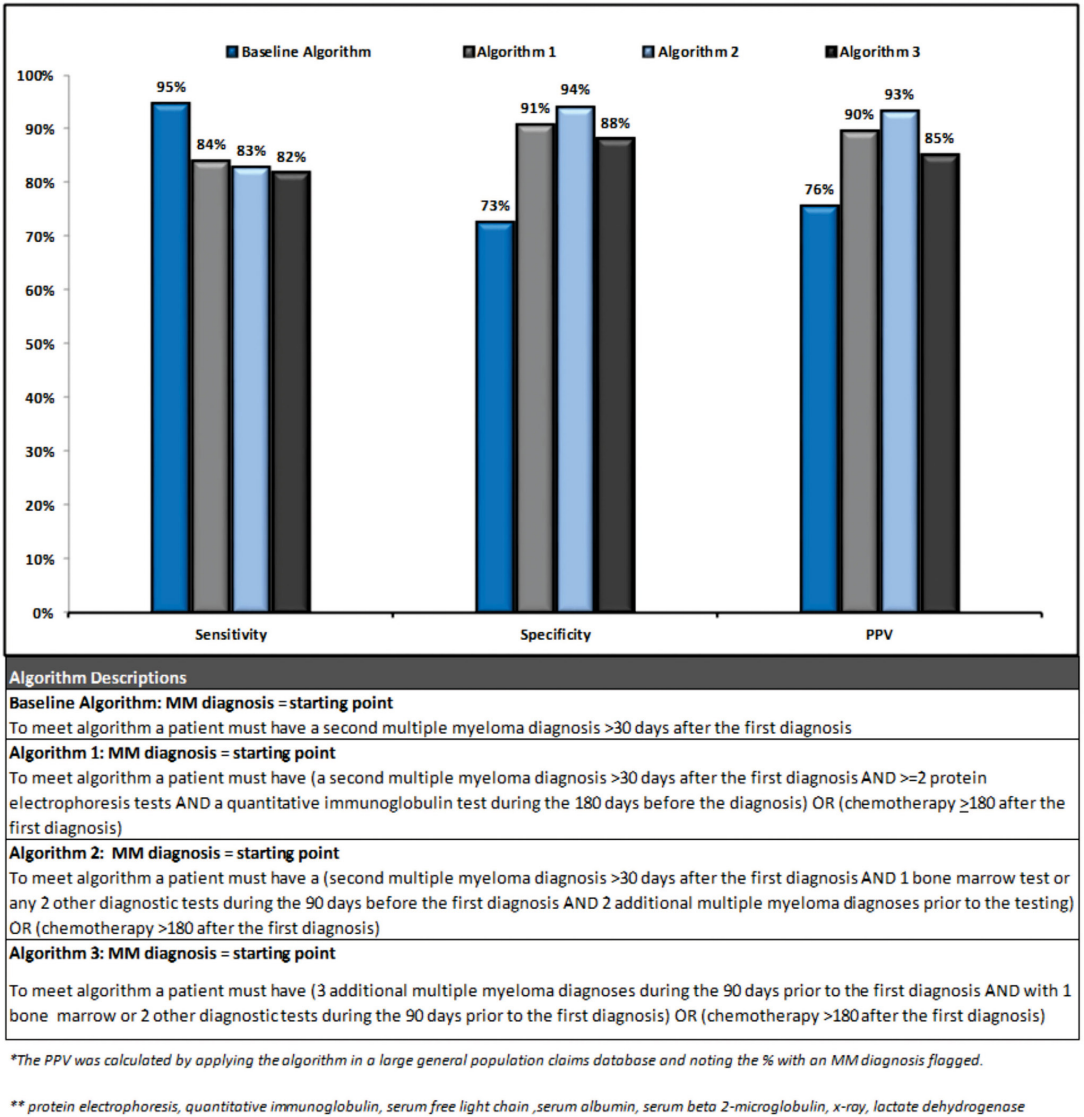
**Algorithm performance results**.

Three out of the four final algorithms had a PPV over 85%, a sensitivity over 80%, and a specificity over 85%. Figure 3 presents a summary of performance and description for each of the algorithms.

The second algorithm had the highest performance (sensitivity 83%, specificity 94%, and PPV 93%) as its increased complexity lead to improvement in specificity. This algorithm identified patients as follows: starting from a MM diagnosis if a patient had ≥1 additional diagnosis >30 days following the index diagnosis, and ≥2 diagnostic tests (or 1 bone marrow test) during the 90 days prior to the index diagnosis, and ≥2 additional MM diagnoses prior to the tests, or chemotherapy during the 180 days following the index diagnosis they were identified as a MM patient. Due to the requirement of 2 MM diagnoses prior to diagnostic testing, this algorithm may be more likely to select a prevalent population and/or a treated population with more symptomatic disease. Visual representation of the algorithms validated in this analysis is provided in Supplementary Material. Algorithms were also tested in the untreated case population separately but no notable patterns could be found to differentiate untreated cases from controls.

## Discussion

This study developed and validated three new administrative claims-based algorithms with ~10% improvement in PPV over the baseline algorithm and prior work (18). Consistent with prior work, we found that a combination of multiple diagnoses and tests were needed to confirm a MM patient truly has the disease (18). Chemotherapy treatment was utilized as an “OR” statement in all three algorithms to improve sensitivity but was not a requirement. This method allows for the selection of untreated patients providing an opportunity to select incident cases or those with SMM. Prior work in the MM population using claims data has relied on the selection of a treated population (15, 16). While the strategy of requiring both appropriate ICD-9-CM codes and the disease-specific treatments can better identify true cases, this approach limits the research question to the treated population and excludes patients who either are at an early disease stage who does not yet require treatment or who forgo treatment for personal or medical reasons.

Because earlier treatment of SMM patients may lead to improved prognosis, longer survival, and prevent progression to symptomatic MM (8, 14), identifying SMM patients is important. Our results suggest that developing an algorithm identifying the SMM population is challenging given the lack of symptoms and treatment, and infrequent testing. SMM cases in this study had characteristics very similar to control patients. Incorrect coding and lack of using a validated algorithm to select patients is a common source of misclassification in cancer studies using secondary data sources (1). Results from this study support the need for validated algorithms in patient selection. Control patients had a much higher than expected number of ICD-9-CM diagnosis codes for MM during a time period when they were known to not have the disease, suggesting that using just one or two claims with a diagnosis code is not enough to identify the appropriate population.

There are several limitations to this study. Potential measurement error in selection and labeling of cases as incident or prevalent is possible since patients appear in EMR systems only as long as they visit clinics and have billing records. Care that patients receive outside the clinic cannot be captured. These challenges of incomplete records and measurement error inherent in EMR databases must be considered when using such data. Second, cases and controls were identified by two different EMR systems (Oncology and Primary Care), and there may be variability in the completeness of data or how information is entered and linked across systems. Additionally, the percentage of patients flagged in the MarketScan Commercial and Medicare databases for the PPV calculations was influenced by the duration of time used to identify potential patients. These algorithms should be implemented and validated in additional data sources to analyze robustness.

Identification of MM prior to the development of symptomatic disease presents a number of challenges due to the complex diagnostic process. Using a validated algorithm, such as the three presented in this analysis, with adequate sensitivity, specificity, and PPV is important when using a secondary data source. Each of the algorithms developed and validated have strengths (i.e., higher specificity vs. higher sensitivity) and variations in complexity that should be considered when selecting which one to use for an analysis. Further research is needed in additional databases to further investigate identifying patients with SMM. Although the algorithms validated in this analysis allow for identification of an incident population, they were still more likely to identify patients after symptoms appeared.

## Author Contributions

NP, CG, TW, MM, DF, WW, GL, and KF all met the four criteria for authorship as listed below: substantial contributions to the conception or design of the work; or the acquisition, analysis, or interpretation of data for the work; and drafting the work or revising it critically for important intellectual content; and final approval of the version to be published; and agreement to be accountable for all aspects of the work in ensuring that questions related to the accuracy or integrity of any part of the work are appropriately investigated and resolved.

## Conflict of Interest Statement

NP, GL, TW, KF, and CG are/were employees of Truven Health Analytics at the time in which the project was completed, which was paid by Onyx Pharmaceuticals Inc., an Amgen subsidiary in connection with the research and development of this manuscript. WW, MM, and DF were all employees of Onyx Pharmaceuticals Inc., an Amgen subsidiary at the time in which the project was completed.
